# Joint Bayesian modeling of time to malaria and mosquito abundance in Ethiopia

**DOI:** 10.1186/s12879-017-2496-4

**Published:** 2017-06-12

**Authors:** Denekew Bitew Belay, Yehenew Getachew Kifle, Ayele Taye Goshu, Jon Michael Gran, Delenasaw Yewhalaw, Luc Duchateau, Arnoldo Frigessi

**Affiliations:** 1 School of Mathematical and Statistical Sciences, College of Natural and Computational Science, Hawassa University, Hawassa, Ethiopia; 2 Department of Statistics and Operations Research, University of Limpopo, Limpopo, South Africa; 3 Oslo Center for Biostatistics and Epidemiology, University of Oslo and Oslo University Hospital, Oslo, Norway; 4 Department of Medical Laboratory Sciences and Pathology, College of Health Sciences, Jimma University, Jimma, Ethiopia; 5 Department of Comparative Physiology and Biometrics, Ghent University, Ghent, Belgium

**Keywords:** Mosquito abundance, Time to malaria, MCMC, Abundance and incidence interaction, Bayesian inference

## Abstract

**Background:**

This paper studies the effect of mosquito abundance and malaria incidence in the last 3 weeks, and their interaction, on the hazard of time to malaria in a previously studied cohort of children in Ethiopia.

**Methods:**

We model the mosquito abundance and time to malaria data jointly in a Bayesian framework.

**Results:**

We found that the interaction of mosquito abundance and incidence plays a prominent role on malaria risk. We quantify and compare relative risks of various factors, and determine the predominant role of the interaction between incidence and mosquito abundance in describing malaria risk. Seasonal rain patterns, distance to a water source of the households, temperature and relative humidity are all significant in explaining mosquito abundance, and through this affect malaria risk.

**Conclusion:**

Analyzing jointly the time to malaria data and the mosquito abundance allows a precise comparison of factors affecting the spread of malaria. The effect of the interaction between mosquito abundances and local presence of malaria parasites has an important effect on the hazard of time to malaria, beyond abundance alone. Each additional one km away from the dam gives an average reduction of malaria relative risk of 5.7*%*. The importance of the interaction between abundance and incidence leads to the hypothesis that preventive intervention could advantageously target the infectious population, in addition to mosquito control, which is the typical intervention today.

## Background

Malaria has an estimated incidence of more than 300 million new cases every year world wide. About one million people die each year due to malaria, of which most of them are in sub-Saharan Africa [1–4]. Approximately 90% of the malaria cases are related to environmental factors [5]. In Ethiopia three-fourths of the land below 2000 meters of altitude is malarious, with two-thirds of the country’s population at risk of malaria infection. In Ethiopia alone there is an average of 5 million cases a year, causing 70,000 deaths each year and accounting for 17% of outpatient visits to health institutions [6]. It has been argued that economic growth is dependent on two key factors, namely the timely arrival of seasonal rain fall and malaria epidemics [7–9].

The establishment and operation of water resource development projects represents an important risk factor, since dams and irrigation schemes transform ecosystems and can substantially change the nature of malaria risk proximal to their location. There is a substantial body of literature documenting the increases in malaria incidence as a consequence of such projects [3]. Ethiopia has recently constructed a large number of dams to produce electricity and for irrigation [10, 11]. Even though dams give economic benefits, they can increase the survival, density and distribution of disease vectors transmitting parasites such as malaria, by providing appropriate habitats. In the region of Ethiopia where this study has been conducted, *Anopheles gambiae sensu lato* is the vector of the malaria parasites, *Plasmodium falciparum* and *Plasmodium vivax*, which occur in the blood of humans who are affected by malaria, during clinical episodes of the disease.

The data in this study originate from a project which aimed to assess the effect on malaria incidence [10] of the construction of a mega hydropower dam in Gilgel Gibe in southwest Ethiopia. A cohort of healthy children was followed over two years, recording the first malaria episode for each cohort of children. Important covariates are the shortest distance of households to the dam shore, seasonal precipitations, temperature and relative humidity. Mosquito counts were also collected in each village, see below for details.

In this paper we explore *jointly* the association between longitudinal measurement of mosquito abundance and time to malaria in the same cohort of children. We investigate the effect of distance, seasonal precipitations, temperature, structure of the house and relative humidity in a mixed effect model for the measured mosquito abundance. This allows us to define a latent variable of mosquito abundance in every village which is more regular than the measured abundance. These latent variables enter into a proportional hazard model for the time to malaria data, together with their interactions with an approximated measure of the time varying malaria incidence in each village. The two models are estimated jointly using an iterative procedure (Markov Chain Monte Carlo) developed and implemented in [12]. Our approach follows a Bayesian version of the joint modeling of longitudinal and time to event data [13].

We investigate whether the distances of households to the dam and the seasonal factors are significantly associated to mosquito abundance. We study the effect of the regularised mosquito abundance in the malaria hazard model, and quantify the role of its interaction with the measure of malaria incidence village-wise on malaria risk.

Previously, Getachew et al. [10] used two separate models, a mixed Poisson regression model for the mosquito abundance and a frailty model for the time to malaria. They found that distance of households to the dam was not significantly associated to malaria incidence, while seasonal effects were significantly associated with malaria incidence. In the paper [11] the authors investigated further the effect of distance of households from the dam on Anopheles mosquito abundance. They found that the abundance was varying significantly between seasons and was higher in villages near the dam.

### Gilgel Gibe data

**Fig. 1:**
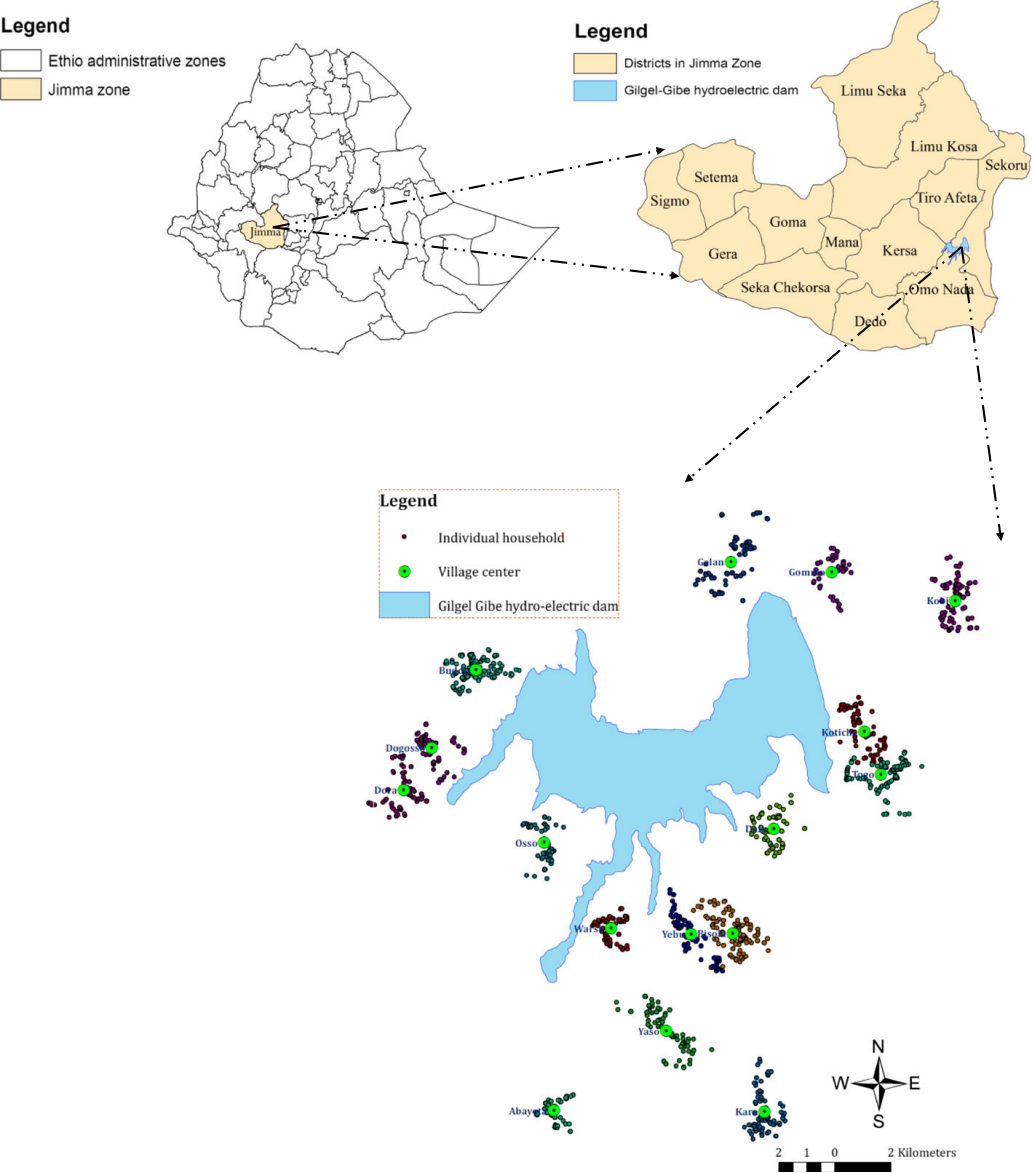
Map of Gilgel Gibe Dam. Gilgel Gibe hydro-electric dam reservoir together with the location of study households and villages around the dam, reproduced from [11] with permission

In this paper we analyse the data previously studied in [10]. The study area is in southwest Ethiopia, around the Gilgel Gibe hydro-electric dam, which created an artificial lake. Sixteen villages were part of the study, in the four districts Sekoru, Tiro afeta, Omo nada and Kersa. The Gilgel Gibe hydro-electric dam area, together with the location of the study households in sixteen villages around the dam are shown in Fig. 1, taken from [11]. The sixteen villages were selected at various distances from the closest dam shore, ranging from 0.265 to 9.056 km. The study period was 23 months long, between 2008 and 2010. In each village on average 130 households were sampled, and in each household one healthy child of age below ten years was randomly chosen and followed from study start. Twenty-nine (1.4%) children died due to various reasons (possibly also malaria related) and 15 (0.72%) migrated elsewhere during the study. Of the migrant children, two children were tracked back and 13 were lost to follow up and excluded from the analysis.

Each child in the cohort of 2040 children was visited weekly during the 23 months of the study and each child was monitored by a trained data collector, who measured his or her body temperature. Parents were asked if the child had signs of fever in the preceding week. In either case, blood from the finger of the child was collected and sent for laboratory analysis to Jimma University referral hospital. There, a blood test was performed to establish if the child had contracted malaria or not. The presence of laboratory confirmed malaria fever is defined as the event in this study. Children who contracted malaria were treated as necessary. Censoring is only related to children who were event free at the end of the study. Age and gender of each child were recorded, together with other variables related to the household. The distance of each household in the study to the closest dam shore was calculated using GPS coordinates.

In the study, an estimate of the prevalence of malaria transmitting mosquitoes has been obtained in the following way. In each village, two houses, one in the center and the other in the periphery of each village, were selected. In each such house mosquitoes were trapped and counted during one night every month using light trap catches from 6pm to 6am. The counts range from 0 mosquitoes to 280 per night in our data. In order to assign a single mosquito abundance estimate per village, we took the average of the two counts per village. This estimated abundance, considered as the exposure to mosquitoes, is therefore the same for all the houses in a village within each month.

Seasonal variability in mosquito abundance is well known, due to varying precipitation, humidity and temperature. Daily rainfall (in mm), monthly average temperature (in °C) and monthly average relative humidity (in percentage) for the whole area have been obtained from the south-western branch regional office of the Ethiopian Meteorological Agency, and are shared by all villages. Finally the way houses are build can also play a role, in particular if they are traditionally build with mud and stick or thatch walls or with corrugated iron. See [4] and [10] for more details.

## Methods

We first perform a simple exploratory data analysis, to investigate visually relations between new malaria cases, rain and mosquito abundance data. We then study the association between the longitudinal measurements and the event of interest through a Bayesian joint model. The mosquito abundance represents the longitudinal component, while the time to malaria is the survival part of the joint model. Further explanations on the derivation of the joint models can be found in the book [13].

### Longitudinal component for anopheles mosquitoes abundance

The time unit in our models is the week *t*. Let *x*_*i*_(*t*) be the measured abundance of mosquitoes at week *t* for child *i*, as measured in her/his village. As abundance data were measured once every month, *x*_*i*_(*t*) is constant over four week periods. Let *k*(*i*)∈1,2,…,16 indicate the village of child *i*. We assume

1 $$ x_{i}(t) = N_{k(i)}(t) + \epsilon_{i}(t),   $$

where *N*_*k*(*i*)_(*t*) is a real valued latent process describing a regularized version of the mosquito abundance at time *t* in the village *k*(*i*) of child *i*, once a measurement error $\epsilon _{i}(t) \sim {\mathcal {N}} (0, \sigma ^{2})$, independent of the other variables, is subtracted from the actual counts.

Next we define a useful weather related covariate. In Ethiopia there are three yearly seasons, namely Bega (dry season), Kiremt (long rainy season) and Belg (short rainy season), based on the magnitude and distribution of rainfall across the 12 months of the year [11]. In [10] these three seasons where identified for the two years and for the region of southwest Ethiopia in our study as the following seven climate periods:

2 $$ {\begin{aligned} \text{period}(t)= \left\{\begin{array}{ll} 1, &\text{when t} \in \text{[weeks in July] in 2008}\\ 2, & \text{when t} \in \text{[weeks from August to November] in 2008} \\ 3, &\text{when t} \in \text{[weeks from December to March] in 2009}\\ 4, &\text{when t} \in \text{[weeks from April to July] in 2009 }\\ 5, &\text{when t} \in \text{[weeks from August to November] in 2009} \\ 6, &\text{when t} \in \text{[weeks from December to March] in 2010} \\ 7, &\text{when t} \in \text{[weeks from April to June] in 2010}. \\ \end{array}\right. \end{aligned}}  $$

The first and last periods are shorter than others, which last four months. See also [11] about this definition of the seasonal periods.

The daily rainfall (in mm/day) precip(*s*) is available for each day *s*, but we found it useful to use smoothed versions. In our model the best fit, based on comparison of the Deviance Information Criterion (DIC) [14], was obtained when averaging over the above climate periods: we define

$$rain(t) = \frac{1}{\text{number of days in period}(t)} \sum_{s \in \text{period}(t)} \text{precip}(s) $$

In order to smooth the step function *r* *a* *i* *n*(*t*), we approximated it with a natural cubic spline with three degrees of freedom and denote as *S*_1_(*r* *a* *i* *n*(*t*)) and *S*_2_(*r* *a* *i* *n*(*t*)) the two B-spline base function components of *r* *a* *i* *n*(*t*), see [12] and [15] for details. In our data there is one global measure of rainfall shared by all children in all villages, which explains the seasonal pattern of the mosquito abundance. Let *t* *e* *m* *p*(*t*) be the monthly average temperature and *h* *u* *m* *i* *d*(*t*) the monthly average humidity of the month of week *t*. We denote by *c* *o* *r* *r* *u* *g* *a* *t* *e*_*i*_ the variable taking value 1 if the house of child *i* had a corrugate iron roof, 0 otherwise. Finally, let *d* *i* *s* *t*_*k*(*i*)_ be the distance from the center of the village *k*(*i*) of child *i* to the closest dam shore.

For the latent abundance *N*_*k*(*i*)_(*t*) we assume the following random effect model:

3 $$ \begin{array}{r} N_{k(i)}(t) = \beta_{0} + \beta_{1} S_{1}(rain(t)) + \beta_{2}S_{2}(rain(t))+\beta_{3} dist_{k(i)} \\ \beta_{4} temp(t) + \beta_{5} humid(t) + \beta_{6} corrugate_{i} \\ + w_{i0} + w_{i1} S_{1}(rain(t)) + w_{i2}S_{2}(rain(t)), \end{array}  $$

where *β*=(*β*_0_,*β*_1_,*β*_2_,*β*_3_,*β*_4_,*β*_5_,*β*_6_) is the vector of fixed effects and *w*_*i*_=(*w*_*i*0_,*w*_*i*1_,*w*_*i*2_) is the vector of random coefficients with three component. We assume that *w*_*i*_ is a priori normally distributed with zero means and covariance matrix *D*

$$w_{i} = (w_{i0}, w_{i1}, w_{i2}) \sim \mathcal{N}_{3} (0, D), $$

where *D* is specified below. We also tested a model without the distance as covariate.

### Time to event model component for malaria contraction

Let *T*_*i*_ denote the time (in weeks) to malaria infection for child *i*, which can be censored at the study end point in week 100. We used a Cox proportional hazard model [16], where the hazard of child *i* at time *t* is

4 $$  \begin{aligned} h_{i}(t)&=h_{0}(t)\exp \left\{ \theta_{1} age_{i}+ \theta_{2} gender_{i}+\alpha_{1} { N_{k(i)}(t)}\right.\\ &\qquad\qquad\qquad\left.+ \alpha_{2} N_{k(i)}(t)I_{k(i)}(t) \right\}. \end{aligned}  $$

Here *a* *g* *e*_*i*_ is the age of child *i* at week one, *g* *e* *n* *d* *e* *r*_*i*_ the gender of the child and the covariate *I*_*k*(*i*)_(*t*) is the total number of new malaria cases in the village *k*(*i*) of child *i* in the last three weeks before and including week *t*. This is a proxy for a local incidence of malaria because the infectious period of malaria infected children lasts approximately 3 weeks. The term *N*_*k*(*i*)_(*t*)*I*_*k*(*i*)_(*t*) represents the interaction between mosquito abundance and incidence. We also tested separately the incidence with a two weeks windows. As suggested in [12], we model the baseline logarithm of the hazard *h*_0_(*t*) with a B-spline with a quite large number of knots, 15 in our analysis, in order to allow for flexibility. Knots are equally spaced according to the percentiles of the observed event times. We assume

$$\log h_{0}(t) = \phi_{0} + \sum_{z=1}^{15}\phi_{z}B_{z}(t,\nu) $$

where *B*_*z*_(*t*,*ν*) denotes the *z* ^*th*^ basis function of a B-spline with knots *ν*=*ν*_1_,*ν*_2_,...,*ν*_15_ and *ϕ* is the vector of all spline coefficients, which are then penalised for smoothness, penalising with the integral of the second derivative of the fitted log hazard, as explained in [12], using a penalty coefficient denoted by *λ*.

### Priors

The prior probability distribution for the fixed effects (*β*_0_,*β*_1_,*β*_2_,*β*_3_,*β*_4_,*β*_5_,*β*_6_) of the linear mixed effects model is assumed to be normal with means equal to zero and a seven by seven precision matrix, i.e. the inverse of the covariance matrix, with diagonal elements equal to 0.01 and zero otherwise.

The prior probability distribution for the regression coefficients (*θ*_1_,*θ*_2_) of the survival model in (4) is assumed normal with mean zero and a 2 by 2 precision matrix with diagonal 0.01 and zero otherwise.

A normal probability distribution prior is also assumed for the association parameters (*α*_1_,*α*_2_) in the survival model, again with means equal to zero and a 2 by 2 precision matrix with diagonal elements 0.1 and zero otherwise.

For the parameters of the log baseline hazard function we follow [17] and assume for the coefficients *ϕ* of the B-spline an improper prior and for the smoothing parameter *λ* a Gamma hyperprior as follows:

$$P(\phi| \lambda) \propto \lambda^{\frac{r(A)}{2}}\exp\bigg(-\frac{\lambda}{2}\phi^{T}A\phi \bigg) $$

where $A = \Delta _{2}^{T}\Delta _{2}$, where *Δ*_2_ is the order two difference penalty matrix and *r*(*A*) denotes the rank of *A*. For the smoothing parameter *λ* we assume a Gamma (1,0.005) prior, which leads to a proper posterior for *ϕ* ([17]). More detailed information can be found in [12, 15].

The prior for the precision matrix *D* ^−1^ of the the random effects in the linear mixed model is a Wishart distribution, because it is the conjugate prior for the precision matrix in Gaussian models [18]. For the hyperprior we performed a preliminary run of the *lme*-function in *R* for model (1) and (3) to obtain an estimate of *D* ^−1^, which we used to fix the scale matrix in the prior as follows:

$$D^{-1} \sim Wish\left(\left[\begin{array}{lll} 53903.4 & 1980.7 & -1096.7\\ 1980.7 & 75.9 & -42.1\\ -1096.7 & -42.1 & 25.6\end{array}\right], 3\right).$$

We also used this scale matrix as starting point of the MCMC, to speed up convergence.

A similar technique was used for the prior probability distribution of the precision parameter (*σ* ^2^)^−1^. This was assumed to be Gamma distributed with hyper-parameters obtained by running the *lme*-function for the mixed effect model (1) and (3). The idea of using a preliminary run in order to fix hyperparameters in the prior in an empirical Bayesian fashion, has been suggested in [12], where also a further general discussion on the choice of priors in the Bayesian joint model is given. See also [19].

### Implementation

We used the R-package JMBayes [12] for our analysis. It implements a full scale MCMC algorithm to sample all parameters in the posterior model. Briefly, the first step is to run the linear mixed effect model on its own and the Cox proportional hazard model with only gender and age as covariates, in order to obtain good starting values for most parameters in the MCMC. Then a Metropolis Hasting algorithm is started, with random walk proposals. Convergence of the MCMC was tested using trace plots and checking robustness of estimates when repeating the runs with different random starting points. We concluded that it was appropriate to run 200,000 iterations in our analysis, after a burn-in of 120,000 iterations. For further details on the algorithm see [12]. Credible intervals are used to describe the variability of the posterior around the posterior mean.

## Results

**Fig. 2:**
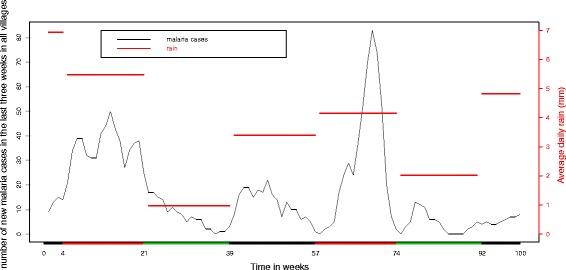
Malaria cases in the last three weeks Vs rain. Total number of malaria cases in the past three weeks before week t in all villages (*left axis*) and average of daily rain(mm) (*right axis*). On the x-axis the colours identify the three seasons: dry season (*green*), long rainy season (*red*), short rainy season (*black*)

In Fig. 2, we plot the total new malaria cases in the last three weeks in all villages together (left axis). This shows a clear association between our measure of incidence and the seasonal rain pattern (right axis). In the main rainy season (coded red in the x-axis), malaria incidence is higher. In the short rains season (black) the incidence is higher than in dry season (green) when incidence is low.

**Fig. 3:**
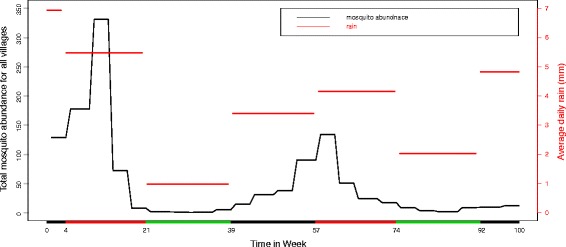
Total mosquito abundance in the past four weeks Vs rain. Total mosquito abundance in the past four weeks before week *t* in all villages (*left axis*) and average of daily rain (mm) (*right axis*). On the x-axis the colours identify the three seasons: dry season (*green*), long rainy season (*red*), short rainy season (*black*)

In Fig. 3 we show the total mosquito abundance (left axis) in all villages as a function of time and average daily rain (right axis). We see that abundance is low in the dry season and builds up during the short rainy season (black) and main rainy season (red). The highest abundance comes typically early in the rainy season, and then it decreases, which might be due to heavy rain flooding mosquito breeding habitats.

Based on the Bayesian joint model we obtain the posterior mean of the parameters in the model with 95% credible intervals, as given in Table 1. The association parameters *α*_1_ and *α*_2_ are different from zero with high posterior probability, as the 95% credible interval does not contain zero. This confirms the utility of the joint model for the present data. The coefficient *α*_1_ is the effect of mosquito abundance on the malaria hazard and has a posterior mean of 0.14, with 95% credible interval (0.06, 0.21). For each unit increase in abundance, assuming all other variables constant, the hazard increases by a factor of exp(0.14)=1.15. The coefficient *α*_2_ controls the effect of the interaction between abundance and local incidence (defined by *I*_*k*(*i*)_(*t*)) and has a posterior mean equal to 0.31 (0.20, 0.41). This indicates that the interaction *N*_*k*(*i*)_(*t*)*I*_*k*(*i*)_(*t*) has an important effect on the hazard of malaria, beyond abundance *N*_*k*(*i*)_(*t*) alone. This is of course reasonable, as both the vector and the parasite must be present together for malaria to occur and spread. As age and gender are not significant (as found in previous studies), the posterior expected hazard can be assumed to be proportional to

5 $$  \begin{aligned} &\text{posterior expected hazard} \propto\\ &\quad\exp \left \{ 0.14N_{k(i)}(t)+ 0.31N_{k(i)}(t)I_{k(i)}(t) \right \}. \end{aligned}  $$

**Table 1:** Parameter estimates and 95% credible intervals for the joint model

	Parameter	Posterior mean	2.5%	97.5%
Abundance model				
Intercept	*β*_0_	3.12	2.89	3.36
*S*_1_(*r* *a* *i* *n*(*t*))	*β*_1_	5.99	5.72	6.26
*S*_2_(*r* *a* *i* *n*(*t*))	*β*_2_	0.67	0.59	0.74
Distance	*β*_3_	-0.13	-0.17	-0.11
Temperature	*β*_4_	-0.15	-0.16	-0.15
Relative humidity	*β*_5_	0.0004	0.0001	0.0006
Corrugate roof	*β*_6_	0.05	-0.07	0.17
Measurement error	*σ*	0.69	0.69	0.70
Time to event model				
Age	*θ*_1_	0.01	-0.03	0.05
Gender	*θ*_2_	-0.05	-0.21	0.12
Association main effect	*α*_1_	0.14	0.06	0.21
Association interaction	*α*_2_	0.31	0.20	0.41
Hyper-parameters				
Penalty	*λ*	0.0031	0.0014	0.0055
Random effect covariance	*D*_1,1_	26.96	25.37	28.70
Random effect covariance	*D*_2,1_	0.75	0.35	1.16
Random effect covariance	*D*_3,1_	-0.80	-1.07	-0.53
Random effect covariance	*D*_2,2_	3.05	2.84	3.27
Random effect covariance	*D*_3,2_	0.82	0.71	0.93
Random effect covariance	*D*_3,3_	1.40	1.31	1.50
DIC	398866.1			

*D*_*i*,*j*_ denotes the *ij*-element of the covariance matrix for the random effects. We use a three week window to define the incidence *I*_*k*(*i*)_(*t*)

This shows that the interaction between the mosquito abundance *N*_*k*(*i*)_(*t*) and the local presence of the malaria parasite, for which *I*_*k*(*i*)_(*t*) is a proxy, is the most important factor. The quantification of this effect is interesting: the hazard increases by a factor of exp(0.14)=1.15 per unit increase of the abundance because of the first term in (5), while it increases by exp(0.31×*I*_*k*(*i*)_(*t*)) because of the second term in (5). As *I*_*k*(*i*)_(*t*) takes values between 0 and 12 across all villages, with mean 1.01, this relative risk is on average exp(0.31×1.01)=1.37, but can reach values as high as exp(0.31×12)=41.26.

The distance to the dam has a negative effect on abundance: the mosquito abundance decreases on average by 0.13 per kilometer away from water keeping other covariates constant. Consequently, the average hazard of time to malaria can be computed as exp(0.14×(−0.13)+0.31×(−0.13)×1.01)=0.943, where we used 1.01 which is the average local incidence in the last three weeks across all villages. This indicates an average reduction of 5.7*%* of the malaria relative risk per km away from the dam.

**Fig. 4:**
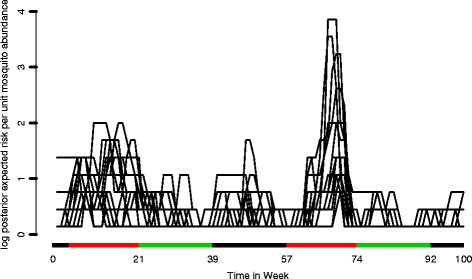
Log posterior expected risk for all villages. The log posterior expected risk of malaria infection for the children in each village per unit increase of mosquito abundance. On the x-axis the colours identify the three seasons: dry season (*green*), long rainy season (*red*), short rainy season (*black*)

In Fig. 4, we plot the log posterior expected risk *α*_1_+*α*_2_ *I*_*k*(*i*)_(*t*)=0.14+0.31*I*_*k*(*i*)_(*t*) per unit mosquito abundance in all villages as a function of time. We can see that there is a seasonal variation in the risk of malaria infection per unit mosquito abundance in all villages. In particular it is higher in the second and the fifth periods, corresponding to the two main rainy seasons in the two years of the study. Beside this, the short rain seasons (Belg) from April to July also show a relatively higher risk of malaria. We also notice in Table 1 that the intercept is significant. This means that our covariates do not explain the mosquito abundance in full and that there is an overall abundance, present constantly and independently of meteorological covariates and distance. To a large extent, this is probably attributable to the fact that our covariates come with large measurement error.

**Fig. 5:**
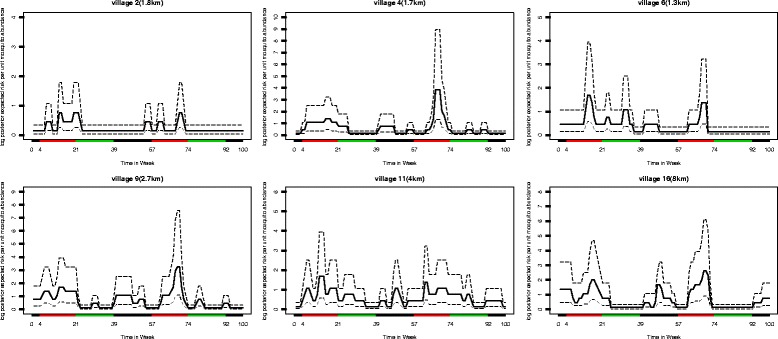
Log posterior expected risk for selected villages. The log posterior expected risk of malaria infection (in *black* colour) and credible intervals (*upper* and *lower boundaries* *dashed*) for the children for a unit increase of mosquito abundance for six villages, with distance to the dam in kilometers. On the x-axis the colours identify the three seasons: dry season (*green*), long rainy season (*red*), short rainy season (*black*)

Figure 5 shows the log posterior expected risk of malaria for six typical villages. We see that in all villages there is a high risk of malaria in the two rainy seasons, but there is a higher variability in the effect of the season between villages. We found that there is more dependence on season than on distance as also discussed in [11]. When the temperature increases, mosquito abundance has a tendency to decrease, similarly with the findings in [11]. Humidity is positive correlated to mosquito abundance, although the effect is minor.

Next we test if a reduction of the weather related covariates would lead to a better model. In Table 2, we use only the rain covariate and not temperature and humidity, nor roof construction. We use the Deviance Information Criterion (DIC) [14] to compare models. The DIC is larger, favouring the use of all covariates. We also tried to drop the distance to the dam as covariate, see Table 3. Again the DIC indicates that the model including the distance and the weather covariates is to be preferred.

**Table 2:** Parameter estimates and 95% credible intervals for the joint model

	Parameter	Posterior mean	2.5%	97.5%
Abundance model				
Intercept	*β*_0_	0.66	0.40	0.91
*S*_1_(*r* *a* *i* *n*(*t*))	*β*_1_	4.40	4.18	4.61
*S*_2_(*r* *a* *i* *n*(*t*))	*β*_2_	0.92	0.85	0.99
Distance	*β*_3_	-0.19	-0.23	-0.12
Measurement error	*σ*	0.71	0.70	0.71
Time to event model				
Age	*θ*_1_	0.01	-0.03	0.05
Gender	*θ*_2_	-0.04	-0.21	0.12
Association main effect	*α*_1_	0.12	0.04	0.19
Association interaction	*α*_2_	0.27	0.16	0.37
Hyper-parameters				
Penalty	*λ*	0.005	0.002	0.008
Random effect covariance	*D*_1,1_	24.22	22.78	25.77
Random effect covariance	*D*_2,1_	0.94	0.60	1.27
Random effect covariance	*D*_3,1_	-0.73	-0.99	-0.47
Random effect covariance	*D*_2,2_	2.16	2.01	2.31
Random effect covariance	*D*_3,2_	0.81	0.72	0.91
Random effect covariance	*D*_3,3_	1.41	1.32	1.51
DIC	403478.6			

*D*_*i*,*j*_ denote the *ij*-element of the covariance matrix for the random effects. Here rain is the only weather related covariate

**Table 3:** Parameter estimates and 95% credible intervals for the joint model

	Parameter	Posterior mean	2.5%	97.5%
Abundance model				
Intercept	*β*_0_	0.18	-0.03	0.39
*S*_1_(*r* *a* *i* *n*(*t*))	*β*_1_	4.37	4.16	4.59
*S*_2_(*r* *a* *i* *n*(*t*))	*β*_2_	0.92	0.85	0.98
Measurement error	*σ*	0.71	0.70	0.71
Time to event model				
Age	*θ*_1_	0.01	-0.03	0.05
Gender	*θ*_2_	-0.05	-0.22	0.12
Association main effect	*α*_1_	0.12	0.04	0.19
Association interaction	*α*_2_	0.26	0.16	0.36
Hyper-parameters				
Penalty	*λ*	0.003	0.002	0.006
Random effect covariance	*D*_1,1_	23.55	22.15	25.05
Random effect covariance	*D*_2,1_	0.58	0.26	0.89
Random effect covariance	*D*_3,1_	-0.73	-0.99	-0.49
Random effect covariance	*D*_2,2_	2.16	2.01	2.31
Random effect covariance	*D*_3,2_	0.81	0.72	0.91
Random effect covariance	*D*_3,3_	1.42	1.32	1.52
DIC	403452.2			

*D*_*i*,*j*_ denotes the *ij*-element of the covariance matrix for the random effects. We use a three week window to define the incidence *I*_*k*(*i*)_(*t*). In this run, the distance is not included in the model

To study robustness of our results with respect to the definition of the incidence, we report in Table 4, the results for the full model with all three weather related covariates, roof construction and the distance, when we only change the definition of *I*_*k*(*i*)_(*t*) to include only the last two weeks (instead of three). Estimates are in practice unchanged, with a slightly larger effect of the distance. The DIC is essentially unchanged, with a slight preference for the three weeks definition of the village-wise incidence. Finally in Table 5, we tested the model with the same definition of incidence, but using only rain as weather covariate and distance. The DIC is again larger.

**Table 4:** Parameter estimates and 95% credible intervals for the joint model

	Parameter	Posterior mean	2.5%	97.5%
Abundance model				
Intercept	*β*_0_	3.28	3.04	3.52
*S*_1_(*r* *a* *i* *n*(*t*))	*β*_1_	6	5.73	6.27
*S*_2_(*r* *a* *i* *n*(*t*))	*β*_2_	0.67	0.59	0.74
Distance	*β*_3_	-0.19	-0.20	-0.17
Temperature	*β*_4_	-0.15	-0.16	-0.15
Relative humidity	*β*_5_	0.0004	0.0002	0.0006
Corrugate roof	*β*_6_	0.05	-0.07	0.17
Measurement error	*σ*	0.69	0.69	0.70
Time to event model				
Age	*θ*_1_	0.01	-0.03	0.05
Gender	*θ*_2_	-0.04	-0.21	0.12
Association main effect	*α*_1_	0.14	0.07	0.21
Association interaction	*α*_2_	0.31	0.20	0.41
Hyper-parameters				
Penalty	*λ*	0.0038	0.0017	0.0069
Random effect covariance	*D*_1,1_	27.10	25.46	28.82
Random effect covariance	*D*_2,1_	0.92	0.51	1.33
Random effect covariance	*D*_3,1_	-0.82	-1.10	-0.55
Random effect covariance	*D*_2,2_	3.05	2.85	3.27
Random effect covariance	*D*_3,2_	0.81	0.71	0.92
Random effect covariance	*D*_3,3_	1.40	1.31	1.50
DIC	398876			

*D*_*i*,*j*_ denotes the *ij*-element of the covariance matrix for the random effects. We use a two week window to define the incidence *I*_*k*(*i*)_(*t*)

**Table 5:** Parameter estimates and 95% credible intervals for the joint model

	Parameter	Posterior mean	2.5%	97.5%
Abundance model				
Intercept	*β*_0_	0.67	0.42	0.92
*S*_1_(*r* *a* *i* *n*(*t*))	*β*_1_	4.39	4.18	4.61
*S*_2_(*r* *a* *i* *n*(*t*))	*β*_2_	0.92	0.85	0.99
Distance	*β*_3_	-0.19	-0.23	-0.15
Measurement error	*σ*	0.71	0.70	0.71
Time to event model				
Age	*θ*_1_	0.01	-0.03	0.05
Gender	*θ*_2_	-0.05	-0.21	0.13
Association main effect	*α*_1_	0.12	0.04	0.19
Association interaction	*α*_2_	0.26	0.16	0.36
Hyper-parameters				
Penalty	*λ*	0.004	0.002	0.008
Random effect covariance	*D*_1,1_	24.25	22.81	25.80
Random effect covariance	*D*_2,1_	0.95	0.62	1.28
Random effect covariance	*D*_3,1_	-0.73	-0.99	-0.47
Random effect covariance	*D*_2,2_	2.16	2.01	2.30
Random effect covariance	*D*_3,2_	0.81	0.72	0.91
Random effect covariance	*D*_3,3_	1.41	1.32	1.51
DIC	403463.5			

*D*_*i*,*j*_ denote the *ij*-element of the covariance matrix for the random effects. Here we use a two week window to define the incidence *I*_*k*(*i*)_(*t*). Only rain is used as weather related covariate

The role of distance has been discussed before in [10]. Table 3 reports the results when we use only seasonal rain as covariate in the abundance model (3). Here we use three weeks accumulated cases for *I*_*k*(*i*)_(*t*). The estimates of the remaining parameters are in practice unchanged, which shows that the distance (significant in Table 1) helps explain the abundance component in addition to the other covariates. The main difference between Tables 3 and 1 is that the intercept in the abundance model (3) does not stay away from zero with high posterior probability when the distance covariate is dropped, and the random effect covariances are also smaller. This shows, in regularisation of mosquito abundances using model (3), there is more subject variability in Table 1 than in Table 3, when we use only seasonal rain.

## Discussion

In this paper we propose the use of a Bayesian joint model to integrate time to malaria and mosquito abundance longitudinal data. Our model allows to represent the causal flow in a natural way: distance to the water, seasonal precipitations and other covariates induce mosquito abundance, which in turn by interacting with the presence of malaria parasites, leads to new malaria cases. We found that the two parameters *α*_1_ and *α*_2_ are estimated significantly away from zero. This indicates that the two component of the joint model (the longitudinal and the time to event) are linked, justifying the importance of using a joint model approach.

The estimated hazard (as in Fig. 4), follows the seasonal pattern. The local precipitation, when regularised into appropriate seasons and expressed as average daily rain, is associated with new malaria cases recorded in the last three weeks and accumulated across villages. Also, our measure of mosquito abundance is correlated to average seasonal daily precipitation, with abundance peaking early in the main rainy season. This confirms the finding in [11] that observed that Plasmodium falciparum malaria risk and seasons are significantly associated. Temperature is negatively correlated to mosquito abundance. This can be explained by the increased evaporation of local water basins and it become less favourable for mosquitoes larvae to survive [8]. Humidity had a marginal role in our model, though it was positively associated to mosquito abundance.

Our results allow quantifying and comparing the contributions to malaria risk of various factors. We found that one additional captured mosquito in the sentinel household increased the relative of risk of malaria by 15%. It is of course not easy to interpret the abundance measure used in our data beyond this study, as it is difficult to say what a unit increase of our abundance measure actually means generally. To try to get an idea of this, we observe that in each village, the captured mosquito numbered from zero to 150 per week per village, and were on average 3.2. This gives an indication of how to understand the mentioned 15% increase.

In this study we used as a measure of the village-wise active malaria incidence the number of new children cases in the previous three weeks, as three weeks is the typical length of a malaria episode, during which the parasite is transmittable [20]. We estimate an increase of the relative risk of malaria to be 36% for a unit increase of the product of the abundance times the incidence. This can happen for example if both the mosquito count and the incidence increase by one, but there are of course many others combination (like 0.5 and 2, respectively). Interestingly, the interaction between mosquito abundance and incidence has a larger effect, twice as large, on the hazard as compared to abundance alone. It is expected that this interaction has a larger effect than abundance, as both the parasite and the mosquito need to be present for malaria to spread; however the quantification of the relative risks is interesting.

We tested if other reduced models could perform better than the model with temperature, humidity and rain as weather related covariates and distance to the dam as covariate. We computed the DIC measure to compare models, and found that the full models should be preferred. In particular, the distance appears to be an important covariate in the longitudinal model component, supporting the correct way of using such a distance in modelling malaria risk. Models using the reduced definition of measure of the village-wise active malaria incidence to just the last two weeks (instead than three), were not better. We prefer a three weeks window, due to the three week infectiousness of malaria patients. But if we use the results of that analysis, we found that one additional captured mosquito in the household increased the relative of risk of malaria by 15%. The increase of the relative risk of malaria is now 36*%* for a unit increase of the product of the abundance times the incidence.

The importance of the interaction between abundance and incidence leads us to the hypothesis that preventive intervention could advantageously target the infective population, for example by isolation from the rest of the household during disease activity, in addition to mosquito control, which is the main mean of control today.

The construction of dams in Ethiopia has been documented to result in increases in vector populations by creating mosquito habitats [11, 21, 22]. Several studies have investigated the effect of dams on malaria prevalence and incidence [23–25]. In general, dams can have profound effects on the survival, density, and distribution of disease vectors and parasites by altering the local ecology and habitats, and the altered vector/parasite ecology modifies the transmission of vector borne diseases such as malaria and its incidence [26]. Kibret et al. (2009) investigated the association of Koka reservoir and Anopheles mosquito density and malaria risk. Accordingly, higher Anopheles arabiensis density and malaria incidence was reported from villages in the vicinity of the reservoir. The construction and operation of dams in Senegal River increased anopheline densities, malaria transmission intensity and prevalence was higher in villages closer to dams than in those farther away [27]. A study in Ethiopia also showed that children living in close proximity to the reservoir created by the newly constructed dam were at a greater risk of Plasmodium infection than children living further away [28]. For the Gilgel Gibe cohort, the distance to the dam has not been previously found significant. In our joint model, the distance is a significant factor in the longitudinal mosquito abundance model, and through the coupling of this model to the time to malaria model, it affects the risk of malaria.

Indeed, distance to the dam has a significant effect on the longitudinal mosquito abundance component of the joint model. We can compare the effect of being closer to water and of other risk factors. For example, we showed that for a household to move 1km closer to the water, is (on average) equivalent to capturing 0.13 additional mosquito, which in turn increases the relative risk of malaria by 5.7*%*. Significant association of mosquito abundance with distance to the dam shore improves the results in [10]: we can now show that the distance has an indirect effect on the malaria infection through the mosquito abundance since the breeding takes place in a water body.

We found that the parameter related to the construction of the houses was not estimated significantly off from zero, despite the 95% credible interval was covering a mostly positive range. This tendency confirms what found in [29], namely that people living in Mozambique in houses with grass or thatch roofs had a greater risk of malaria than those living in houses with corrugated iron roofs.

We did not have access to local precipitation data, and instead used a common measure of rain for all villages. We believe that the effect of precipitation on village-wise mosquito abundance would be stronger if we would have precipitation at a finer spatial scale. Future studies should include local measurements of rain, at least weekly. The dam is likely to be important for mosquito reproduction when there are no alternative water basins in and around the villages, as is very often the case in the rainy season in the study area. Therefore we could expect to find distance to the dam to interact with local measures of precipitations, in affecting mosquito abundance. These more precise factors could not be determined with the current data.

The joint model can be used also to predict time to malaria, given forecasts of mosquito abundance and local malaria incidence. While predictions of malaria free survival curves for individual children appeared to have too large credibility bands, we obtained a good accumulated forecasts across all children. However, this experiment was based on actually incurred abundance and incidence and as such it is of limited interest and therefore not reported further in this paper. More work is needed to make the joint model useful for prediction, especially because uncertainties in covariates need to be incorporated. The simulation of each individual child ahead in time, using the joint model in a generative way, is a possibility for further work.

## Conclusions

In this paper we analyzed for the first time the two data components that play a crucial role in the spread of malaria jointly, namely the abundance of the vector and the appearance in time of new malaria cases. We used regional precipitations, temperature and relative humidity as weather covariates, the presence of a corrugated iron roof and the shortest distance to a dam as covariates. The Bayesian joint model appears to be an appropriate method to perform the combined or joint analysis. In this way we can attribute the effect of distance to water and of meteorological covariates to the mosquito vector, while the time to malaria model depends on these covariates only through the abundance of the vector. We found that the interaction between mosquito abundance and incidence plays the key role. Because of the joint analysis, we can compare the contribution to malaria risk of distance to water and mosquito abundance. Further work will explore the possibility to use the joint model to perform prediction of the risk of future infection in the transmission of malaria in the study area.

## Abbreviation

MCMC: Markov Chain Monte Carlo
